# Survey and Analysis of Chemoprophylaxis Policies for Domestic Travel in Malaria-Endemic Countries

**DOI:** 10.3390/tropicalmed7070121

**Published:** 2022-06-29

**Authors:** John Kevin Baird, Marian Warsame, Judith Recht

**Affiliations:** 1 Eijkman-Oxford Clinical Research Unit, Eijkman Institute of Molecular Biology, Jakarta 10430, Indonesia; 2 The Centre for Tropical Medicine and Global Health, Nuffield Department of Medicine, University of Oxford, Oxford OX3 7FZ, UK; 3 School of Public Health and Social Medicine, Institute of Medicine, Gothenburg University, 41390 Gothenburg, Sweden; marian.warsame@gu.se; 4 Independent Researcher, North Bethesda, MD 20852, USA; rechtj@gmail.com

**Keywords:** domestic travel chemoprophylaxis, malaria, tafenoquine

## Abstract

The prevention of malaria in travelers with the use of antimalarials often occurs in connection with international travel to areas of significant risk of infection. Although these travelers sometimes cause outbreaks in their malaria-free home countries, the cardinal objective of prescribed chemoprophylaxis is to protect the traveler from patent malaria during travel. Here we consider the chemoprophylaxis of domestic travelers from malaria-free but -receptive areas within malaria-endemic countries. The main objective in this setting is the protection of those areas from reintroduced malaria transmission. In order to better understand policy and practices in this regard, we surveyed malaria prevention and treatment guidelines of 36 malaria-endemic countries and 2 that have recently eliminated malaria (Sri Lanka, China) for recommendations regarding malaria chemoprophylaxis for domestic travel. Among them, just 8 provided specific and positive recommendations, 1 recommended without specific guidance, and 4 advised against the practice. Most nations (25/38; 66%) did not mention chemoprophylaxis for domestic travel, though many of those did offer guidance for international travel. The few positive recommendations for domestic travel were dominated by the suppressive prophylaxis options of daily doxycycline or atovaquone-proguanil or weekly mefloquine. The incomplete protection afforded by these strategies, along with impractical dosing in connection with the typically brief domestic travel, may in part explain the broad lack of policies and practices across malaria-endemic nations regarding chemoprophylaxis.

## 1. Introduction

Substantial gains against the global burden of malaria occurred between 2000 and 2015 but have since leveled to stable numbers [1,2]. That immovable progress may be explained by several factors, including insufficient human or financial resources, inadequate tools, implementation bottlenecks of proven interventions, strategic gaps, conflict crises, or combinations of those factors [3]. This paper explores one possible strategic gap—chemoprophylaxis—that may encumber progress against endemic malaria, especially that occurring in malaria-endemic countries (MECs) nearing or in the latter stages of the elimination of transmission. Contrary to the conventional view of chemoprophylaxis of malaria in travelers as benefiting primarily international travelers or military personnel, we argue these practices may be leveraged to strategic advantage in domestic malaria control and elimination.

Tremendously varied and complex biologic and geographic characteristics shape the subnational landscapes of malaria transmission for all MECs. However, most will share this important characteristic: a mélange of zones varying from no to high risk of infection [4,5]. Those may be so by natural and stable ecologies defining the absence/presence and paucity/abundance of anopheline mosquito vectors [6]. The key point here is that effective malaria control and elimination work creates unnaturally malaria-free zones that remain, in a biological and ecological sense, receptive to reintroduced malaria transmission [7,8]. Those are zones in which human communities no longer carry malaria parasites despite a natural presence of efficient vector anopheline mosquitoes. Areas such as this have been appropriately characterized as vulnerable to reintroduced malaria transmission, and we refer to these as malaria-receptive areas (MRAs). Malaria control methodologies that modify the natural environment in ways that diminish the presence of specific anopheline mosquito vectors, called species sanitation, have become rarely practiced [9]. The core strategy at work today focuses on the human host, i.e., diagnosis and treatment of the infected and providing protection from biting mosquitoes (nets and insecticides). Those approaches leave mosquito ecologies unaltered and those populations unimpacted.

The presence of an infected and infectious person in an MRA poses a direct threat of reintroducing malaria parasites into those human communities. Setting aside the occasionally important problems of illegal crossings of national borders and legal foreign visitors, the overwhelmingly dominant risk involving MRAs within MECs is ordinary domestic travel. That travel is most often relatively brief or seasonal [10]. When it involves visitation from MRAs to areas of active malaria transmission, the potential for reintroduced endemic transmission occurs [11,12,13,14]. In most settings, the diagnostic screening of people visiting or returning from areas of active malaria transmission within MECs is not practical. Domestic travel is not ordinarily controlled by health authorities, and even if it were, the numbers of people moving on most national scales exceed screening capacities. In Indonesia, for example, tens of millions of people move from MRAs on the island of Java to islands of active malaria transmission and back each year (Elyazar I, personal communication). Moreover, the diagnostics available today would miss latent and subpatent carriers of malaria. Chemoprophylaxis for travelers residing in MRAs may mitigate the substantial risk of domestic travel to endemic zones incurred to the traveler and their community. Targeting high-risk travel within borders for chemoprophylaxis interventions may utilize mapping of human movements within national borders [10,15].

Chemoprophylaxis against malaria in travelers differs from the validating evidence, strategies, practices, and aims of chemoprevention (by presumptive therapy rather than chemoprophylactic regimens) benefiting seasonally or physiologically vulnerable (small children and pregnant women) residents of highly endemic areas [16,17]. Those of chemoprophylaxis derive from decades of experience in protecting international visitors to endemic areas from acute malaria during and after travel [18]. Many national malaria control programs (NMCPs) of MECs have not considered guidance and advocacy for chemoprophylaxis for domestic travel. The 2022 Guidelines for Malaria from the World Health Organization (WHO) also offer no guidance on this practice [19]. Here we report a survey of the malaria prevention and treatment guidelines with specific regard to chemoprophylaxis for domestic travel. We aimed to characterize the extent to which chemoprophylaxis against malaria may be recommended and the character of that guidance. The findings offer context for exploring how chemoprophylaxis for domestic travel from MRAs within national borders of MECs may be improved and implemented.

## 2. Survey

### 2.1. Selection of Malaria-Endemic Countries for Survey

We sampled all WHO regions that included MECs: African, Eastern Mediterranean, Pan American, Southeast Asia, and Western Pacific Regions. We selected nations having zones of active, inactive, or absent malaria transmission. We included nations with accessible English, Spanish, or Portuguese versions or translations of NMCP malaria prevention and treatment guidelines (MPTGs). Table 1 lists the nations surveyed according to WHO regional offices. A total of 38 nations were surveyed.

### 2.2. Malaria Prevention and Treatment Guidelines

We obtained publicly available MPTGs issued by NMCPs from an archive of those maintained by the Global Malaria Program of the WHO and used them for this survey with permission. We also visited the websites of NMCPs to obtain their most recent MPTGs. In some instances, personal contacts linked to NMCPs provided MPTGs or reported to us content relevant to this survey. Most of the MPTGs we examined were dated between 2012 and 2018, but some were dated as far back as 2008 (Bolivia and Brazil) or as recent as 2019 (Nicaragua and Afghanistan). It is acknowledged that some of these may not have been the most recently published MPTGs but were nonetheless suitable for the purpose of this analysis because we expected that chemoprophylaxis recommendations would be much less dynamic across years than treatment guidance for acute malaria.

### 2.3. Classification of Chemoprophylaxis Guidance

We classified each national MPTG according to content expressing guidance relevant to chemoprophylaxis for domestic travel, as listed in Table 2. We extracted specific recommendations, both positive and negative, from those MPTGs offering them. In some instances, nations expressed specific recommendations that were, conditionally, both positive and negative.

## 3. Survey Findings

### 3.1. African Region

Malaria treatment guidelines of 11 countries in the African Region were reviewed to establish recommendations on chemoprophylaxis for domestic travel. The survey showed that eight countries (Angola, Botswana, Cameroon, Ghana, Kenya, Mozambique, Madagascar, and South Africa) did not recommend chemoprophylaxis for domestic travelers (Table 3). However, most of those did explicitly recommend chemoprophylaxis for international travelers, most commonly recommending mefloquine, doxycycline, or atovaquone-proguanil. The remaining three did mention chemoprophylaxis. Ethiopia and Namibia specified chemoprophylaxis for travel to high-risk areas, presumably inclusive of domestic travel. Nigeria recommended unspecified chemoprophylaxis for nonimmune visitors at high risk, again presumably including domestic travelers.

### 3.2. Eastern Mediterranean Region

Table 4 shows the absence of chemoprophylaxis recommendations for domestic travel in the two Eastern Mediterranean Region nations surveyed, Afghanistan and Pakistan. No other countries in that region have areas of endemic transmission that would warrant chemoprophylaxis for any traveler, foreign or domestic. Afghanistan has such areas, as does Pakistan. However, in Pakistan, malaria transmission occurs in large cities due to urbanized *Anopheles stephensi* mosquito populations. Chemoprophylaxis for travel from those cities to malarious rural zones may reasonably be viewed as futile in a public health sense.

### 3.3. Pan American Region

Table 5 lists chemoprophylaxis guidance classifications among the 10 Pan American Region nations surveyed. Two nations (Brazil and Mexico) offered specific and positive recommendations. However, the Brazilian guidance restricted the practice to high-risk *P. falciparum* in remote areas and recommended against its use under other circumstances. The Brazil MPTG specifically mentions the futility of standard chemoprophylaxis against its dominating *P. vivax* problem, presumably referring to post-travel relapses rather than primary attacks while traveling. Mexico recommended standard weekly chloroquine prophylaxis against its endemic *P. vivax* (virtually no *P. falciparum* transmission). None of the eight other nations surveyed mentioned chemoprophylaxis for domestic travel, though some recommended personal protection measures, such as mosquito avoidance by clothing, nets, and repellents.

### 3.4. Southeast Asian Region

Table 6 lists chemoprophylaxis guidance classifications among Southeast Asian Region nations surveyed. Four nations (India, Indonesia, Nepal, and Sri Lanka) of the seven surveyed provided specific recommendations for chemoprophylaxis of domestic travelers. India and Indonesia recommended daily doxycycline, and India also recommended weekly mefloquine. Nepal advised against the practice, while Sri Lanka (currently free of malaria transmission within its borders) referred crossborder travelers to the relevant health authorities to obtain unspecified guidance and medication. Bangladesh mentioned chemoprophylaxis and offered weekly mefloquine but explicitly discouraged its use even in special risk groups. Thailand and Timor-Leste made no mention of chemoprophylaxis for travelers.

### 3.5. Western Pacific Region

Table 7 lists chemoprophylaxis guidance classifications among the eight Western Pacific Region nations surveyed. Three nations (Malaysia, Papua New Guinea, Philippines) provided specific recommendations for chemoprophylaxis for domestic travel. The advice from Papua New Guinea appeared addressed to “inbound travelers” from other nations rather than domestic travelers. Malaysia and Papua New Guinea each recommended daily doxycycline or atovaquone-proguanil, while the Philippines recommended daily doxycycline or weekly mefloquine. Cambodia mentioned chemoprophylaxis but recommended against the practice, citing low risk nationwide. China, Laos, South Korea, and Vietnam made no mention of chemoprophylaxis for travelers.

### 3.6. All Regions

Table 8 summarizes the survey findings. Most of the nations surveyed (25/38; 65%) did not mention chemoprophylaxis for domestic travel. Many of those did mention chemoprophylaxis but offered guidance only for international travel, usually both inbound and outbound or not specified. Four nations (11%; Nepal, Cambodia, Bangladesh, and Brazil) advised against chemoprophylaxis for domestic travel, although Brazil did recommend it for *P. falciparum* risk in remote areas far from care. Eight nations (21%; Ethiopia, Namibia, Mexico, India, Sri Lanka, Indonesia, Malaysia, and the Philippines) offered specific recommendations for chemoprophylaxis with travel to high-risk areas, most of those presumably including within national borders. Nigeria recommended chemoprophylaxis for domestic travel but without offering specific guidance. All recommendations for chemoprophylaxis by these nations in connection with international, domestic, or unspecified travel destinations of high risk involved suppressive chemoprophylaxis drugs, mostly mefloquine, atovaquone-proguanil, or doxycycline.

## 4. Implications

Most nations with endemic malaria do not recommend chemoprophylaxis for domestic travel to high-risk areas. This may, in part, be explained by the lack of the same in the WHO guidelines for managing malaria control and elimination [19]. WHO guidance for travel-associated chemoprophylaxis is found only in its International Travel Health manual [20], and it lists atovaquone-proguanil, mefloquine, and doxycycline as options. These are the same options offered by NMCPs for high-risk travel. It may be reasonably argued that none of these options is suited to the purpose of protecting MRAs from domestic travelers, primarily because all are suppressive rather than causal prophylactics. That is, they act against the plasmodia in the bloodstream rather than earlier in the liver. Although atovaquone-proguanil appears to have causal activity against hepatic schizonts of *P. falciparum*, it does not prevent the formation of latent hypnozoites of *P. vivax* [21,22]. None of those favored options will prevent delayed attacks of relapsing malaria occurring in the weeks and months following travel [23]. Another very significant problem with suppressive chemoprophylaxis for domestic travel from MRAs is prolonged dosing for what is most often brief travel [10]. Mefloquine requires either a large loading dose or several weeks of dosing prior to travel. Post-travel dosing of at least 7 days (atovaquone-proguanil) and as long as 28 days (doxycycline and mefloquine) is required. These regimens would be highly impractical in connection with brief domestic travel. There are very significant pitfalls with recommended suppressive chemoprophylaxis strategies. These may be considered futile with respect to mitigating the specific problem of reintroduced malaria to MRAs. This perspective may explain the dominant policy and practice with respect to chemoprophylaxis for domestic travel within MECs, i.e., none at all.

Nevertheless, there may be little doubt concerning the need for protecting MRAs from domestic travelers. Recent travel is a conspicuous risk factor for malaria acquired internationally that may occasionally result in local outbreaks in otherwise malaria-free nations [24,25,26,27]. Recent studies have explored domestic travel and malaria risk among residents of MRAs or nonendemic areas with MECs. Ahmed et al. [28] conducted a literature review and meta-analysis involving nine MECs in sub-Saharan Africa, finding a pooled odds ratio of 3.8 for recent travel and patent malaria. Lynch et al. [29] found an odds ratio of 6.9 for travel among infected Ugandan residents of highland areas. In Swaziland, Tejedor-Garavito et al. [30] found that 67% of residents acutely ill with malaria had returned from local travels. In the western Kenyan highlands, infection by *P. falciparum* was about twice as likely with recent travel to lowland areas relative to no travel [31]. Gabaldon-Figueira et al. [32] considered domestic travel within Venezuela as a key factor behind the recent rapid expansion of re-established endemic malaria transmission in that nation. Very many outbreaks within MRAs of tremendously varied settings and locations occur [12,33,34,35,36,37]. The historical precedents of nearly eliminated malaria transmission in India [38] and reintroduced endemic malaria transmission on the Korean Peninsula [39] offer compelling examples of the potentially serious consequences of seemingly minor outbreaks within MRAs.

## 5. Conclusions

The NMCPs of many MECs, along with the WHO, have not considered chemoprophylaxis of domestic travelers as a practical and useful means of protecting MRAs. The inadequacy of currently available suppressive regimens for that specific purpose may well explain that strategic weakness. Preventing malaria in domestic travelers may be conspicuously important to gaining and protecting MRAs within MECs, but how this may be accomplished is a difficult technical question. The sterilizing protection of causal prophylaxis may be optimal or even required for this purpose but imposes the serious problem of the hemolytic toxicity of available causal prophylactic drugs (primaquine and tafenoquine, both 8-aminoquinolines) in glucose-6-phosphate dehydrogenase-deficient patients. It is possible that the recommended dosing with tafenoquine, which is hemolytic in those patients, may be in great excess of that needed for effective chemoprophylaxis with brief travel [40]. This should be explored as a possibly pragmatic option for domestic travel from MRAs.

## Figures and Tables

**Table 1:** Nations surveyed among five WHO regions.

African	Eastern Mediterranean	Pan American	Southeast Asian	Western Pacific
Angola	Afghanistan	Bolivia	Bangladesh	Cambodia
Botswana	Pakistan	Brazil	India	China
Cameroon		Colombia	Indonesia	Laos
Ethiopia		Honduras	Nepal	Malaysia
Ghana		Mexico	Sri Lanka	Papua New Guinea
Kenya		Nicaragua	Thailand	Philippines
Mozambique		Panama	Timor-Leste	South Korea
Madagascar		Peru		Viet Nam
Namibia		Suriname		
Nigeria		Venezuela		
South Africa				

**Table 2:** Classification of chemoprophylaxis recommendations in MPTGs surveyed.

Absent	No mention of chemoprophylaxis for domestic travel
Present, Unspecific	Chemoprophylaxis for domestic travel recommended but without guidance
Present, Specific-Negative	Chemoprophylaxis for domestic travel specifically discouraged or explicitly not recommended
Present, Specific-Positive	Chemoprophylaxis for domestic travel recommended, and specific guidance offered

**Table 3:** Survey results for the Africa Region.

Countries	Chemoprophylaxis for Domestic Travel	Recommendation in the Guidelines
Angola	Absent	For international travelers: proguanil, mefloquine, doxycycline, or atovaquone-proguanil
Botswana	Absent	For international travelers: mefloquine or atovaquone-proguanil
Cameroon	Absent	For international travelers: atovaquone-proguanil
Ethiopia	Present, specific-positive	Persons who travel to malaria-endemic areas are at risk of acquiring malaria: mefloquine or atovaquone-proguanil
Ghana	Absent	For international travel: atovaquone-proguanil, doxycycline, or mefloquine
Kenya	Absent	For international travelers: mefloquine, atovaquone-proguanil, or doxycycline
Mozambique	Absent	For international travelers: mefloquine, doxycycline, or atovaquone-proguanil
Madagascar	Absent	For international travel: atovaquone-proguanil
Namibia	Present, Specific-Positive	Antimalarial chemoprophylaxis can be recommended for those traveling to high-transmission settings, particularly those with high-risk exposure and lowered immunity (i.e., pregnant women, children under 5, immunocompromised individuals). Doxycycline and atovaquone-proguanil are the recommended chemoprophylaxis of choice
Nigeria 2015	Present, Unspecific	Malaria chemoprophylaxis is not recommended for individuals living in areas of intense transmission; however, people with sickle cell anemia and nonimmune visitors are expected to be on regular chemoprophylaxis, and these risk categories of patients should be targeted with other preventive interventions, e.g., LLINs
South Africa	Absent	Not mentioned

**Table 4:** Survey results for Eastern Mediterranean Region.

Eastern Mediterranean	MPTG Chemoprophylaxis Guidance	Recommendation
Afghanistan (2019)	Absent	No mention
Pakistan (2020)	Absent	No mention

**Table 5:** Survey results for Pan American Region.

Pan American	MPTG Chemoprophylaxis Guidance	Recommendation
Bolivia	Absent	No mention
Brazil	Present Specific-Negative	Not recommended for most of the country Only recommended for travelers to Amazon region with high risk of *P. falciparum* and where diagnosis and treatment >24 h away
Colombia	Absent	No mention
Honduras	Absent	No mention
Mexico	Present Specific-Positive	Weekly chloroquine on day of arrival and for 6 weeks after return
Nicaragua	Absent	No mention
Panama	Absent	No mention
Peru	Absent	No mention
Suriname	Absent	No mention
Venezuela	Absent	No mention

**Table 6:** Survey Results for Southeast Asia Region.

Southeast Asian	MPTG Chemoprophylaxis Guidance	Recommendation
Bangladesh	Present Specific-Negative	Weekly mefloquine may be used for special risks but discouraged
India	Present Specific-Positive	Daily doxycycline (for travel <6 wk) or weekly mefloquine (for travel >6 wk)
Indonesia	Present Specific-Positive	Daily doxycycline
Nepal (2019)	Present Specific-Negative	Explicitly advises against chemoprophylaxis for domestic travel, offers specific guidance for international travel
Sri Lanka	Present Specific-Positive	Contact authorities to obtain specific recommendations and medication
Thailand	Absent	No mention
Timor-Leste	Absent	No mention

**Table 7:** Survey results for Western Pacific Region.

Western Pacific	MPTG Chemoprophylaxis Guidance	Recommendation
Cambodia	Present Specific-Negative	Not recommended due to low risk
China	Absent	No mention
Laos	Absent	No mention
Malaysia	Present Specific-Positive	Daily doxycycline or atovaquone-proguanil
Papua New Guinea	Absent	For international travelers: doxycycline or atovaquone-proguanil
Philippines	Present Specific-Positive	Daily doxycycline or weekly mefloquine
South Korea	Absent	No mention
Viet Nam	Absent	No mention

**Table 8:** Summary of survey findings.

Region	Nations Surveyed	Absent	Present, Specific-Negative	Present, Unspecific	Present, Specific-Positive
African	11	8	0	1	2
Eastern Mediterranean	2	2	0	0	0
Pan American	10	8	1	0	1
Southeast Asian	7	2	2	0	3
Western Pacific	8	5	1	0	2
All Regions	38	25	4	1	8

## Data Availability

The data reviewed in this report are available in the public domain from individual national malaria control programmes or by request from the Global Malaria Programme, World Health Organization.

## Abbreviations

G6PD	Glucose-6-phosphate dehydrogenase, an enzyme vital to protecting red blood cells from the damage caused by 8-aminoquinoline drugs
LLIN	Long-lasting insecticide-treated net, used to protect people from biting mosquitoes while sleeping
MEC	Malaria-Endemic County, a nation having known active malaria transmission anywhere within national borders
MPTG	Malaria Prevention and Treatment Guidelines, composed and made public by NMCP authorities
NMCP	National Malaria Control Program operated by government authorities at the national level
MRA	Malaria-Receptive Area, a subnational area free of malaria transmission but remaining receptive to it by means of natural anopheline populations
WHO	World Health Organization in Geneva, Switzerland, operating globally through regional offices
